# Cell‐based therapy to reduce mortality from COVID‐19: Systematic review and meta‐analysis of human studies on acute respiratory distress syndrome

**DOI:** 10.1002/sctm.20-0146

**Published:** 2020-05-29

**Authors:** Wenchun Qu, Zhen Wang, Joshua M. Hare, Guojun Bu, Jorge M. Mallea, Jorge M. Pascual, Arnold I. Caplan, Joanne Kurtzberg, Abba C. Zubair, Eva Kubrova, Erica Engelberg‐Cook, Tarek Nayfeh, Vishal P. Shah, James C. Hill, Michael E. Wolf, Larry J. Prokop, M. Hassan Murad, Fred P. Sanfilippo

**Affiliations:** ^1^ Department of Pain Medicine Mayo Clinic Jacksonville Florida USA; ^2^ Center for Regenerative Medicine Mayo Clinic Jacksonville Florida USA; ^3^ Evidence‐Based Practice Center Mayo Clinic Rochester Minnesota USA; ^4^ Robert D. and Patricia E. Kern Center for the Science of Health Care Delivery Mayo Clinic Rochester Minnesota USA; ^5^ Interdisciplinary Stem Cell Institute and Cardiology Division, Department of Medicine University of Miami, Miller School of Medicine Miami Florida USA; ^6^ Department of Neuroscience Mayo Clinic Jacksonville Florida USA; ^7^ Division of Pulmonary, Allergy and Sleep Medicine, Department of Medicine Mayo Clinic Jacksonville Florida USA; ^8^ Skeletal Research Center, Biology Department Case Western Reserve University Cleveland Ohio USA; ^9^ Marcus Center for Cellular Cures Duke University Medical Center Durham North Carolina USA; ^10^ Transfusion Medicine and Stem Cell Therapy, Department of Laboratory Medicine and Pathology Mayo Clinic Jacksonville Florida USA; ^11^ Department of Physical Medicine and Rehabilitation, Department of Orthopedic Surgery Mayo Clinic Rochester Minnesota USA; ^12^ Department of Preventative, Occupational, and Aerospace Medicine Mayo Clinic Rochester Minnesota USA; ^13^ Mayo Clinic Library Rochester Minnesota USA; ^14^ Department of Pathology and Laboratory Medicine, Department of Health Policy and Management Rollins School of Public Health, Emory University, The Marcus Foundation Atlanta Georgia USA

**Keywords:** acute respiratory distress syndrome, COVID‐19, mesenchymal stromal cells, mortality, systematic review

## Abstract

Severe cases of COVID‐19 infection, often leading to death, have been associated with variants of acute respiratory distress syndrome (ARDS). Cell therapy with mesenchymal stromal cells (MSCs) is a potential treatment for COVID‐19 ARDS based on preclinical and clinical studies supporting the concept that MSCs modulate the inflammatory and remodeling processes and restore alveolo‐capillary barriers. The authors performed a systematic literature review and random‐effects meta‐analysis to determine the potential value of MSC therapy for treating COVID‐19‐infected patients with ARDS. Publications in all languages from 1990 to March 31, 2020 were reviewed, yielding 2691 studies, of which nine were included. MSCs were intravenously or intratracheally administered in 117 participants, who were followed for 14 days to 5 years. All MSCs were allogeneic from bone marrow, umbilical cord, menstrual blood, adipose tissue, or unreported sources. Combined mortality showed a favorable trend but did not reach statistical significance. No related serious adverse events were reported and mild adverse events resolved spontaneously. A trend was found of improved radiographic findings, pulmonary function (lung compliance, tidal volumes, PaO_2_/FiO_2_ ratio, alveolo‐capillary injury), and inflammatory biomarker levels. No comparisons were made between MSCs of different sources.

## Graphical abstract

In ARDS, pro‐inflammatory cytokines contribute to the disruption of the alveolar – capillary membrane, edema, and pneumocyte damage. MSCs modulate the inflammatory milieu by T‐cell and macrophage redirection, and anti‐inflammatory cytokine production. Clinical trials assessing MSC therapy in ARDS have shown that MSCs have been associated with a favorable trend in morality reduction, pulmonary function improvement, and cytokine correction.

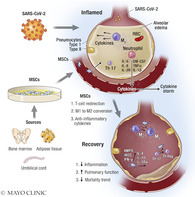

**Significance statement**

The potential benefits of mesenchymal stromal cell (MSC) therapy for patients with COVID‐19 acute respiratory distress syndrome support the rapid commencement of clinical trials, as well as the compassionate use of MSCs that already have documented safety profiles from FDA‐approved studies.

## 1 INTRODUCTION

As a common enveloped RNA virus that crosses species,^1^, ^2^ the coronavirus has become a source of highly lethal infections in the 21st century, including Severe Acute Respiratory Syndrome (SARS), Middle East Respiratory Syndrome (MERS), and the current pandemic with COVID‐19 (Severe Acute Respiratory Syndrome Coronavirus 2, SARS‐CoV‐2). COVID‐19 has been associated with an intensive care unit (ICU) admission rate of 5% of proven infections^3^ and an overall mortality rate in the range of 0.5% to 7%.^4^ Among patients who require hospitalization, mortality may be approximately 5% to 15%.^3^, ^5^ Current treatment for patients with acute lung injuries is supportive, but with a high case fatality rate of 22% to as high as 88% for ICU patients.^3^, ^5^, ^6^ Importantly, age represents a major risk factor for mortality.^6^ New treatment modalities are needed to save lives by addressing the underlying pathophysiological processes that prevent oxygen exchange and destruction of the alveoli.

Similar to prior findings in SARS and MERS, COVID‐19/SARS‐CoV‐2 in severe cases leads to fatal acute respiratory distress syndrome (ARDS), associated with monocyte and macrophage infiltration, diffuse alveolar damage, and cellular fibromyxoid exudates^7^, ^8^ with mortality reported as high as 52.4%.^4^ The respiratory distress peaks at 7 to 10 days with manifestations of immune dysregulation, including cytokine release syndrome with elevation of cytokine levels (IL‐6, IL‐8, IL‐1, IL2R, IL‐10, and TNF‐α), lymphopenia (in CD4+ and CD8+ T cells), and decreases in IFN‐γ expression in CD4+ T cells.^8^, ^9^ The inverse correlation between cytokine storm with lower CD4+ and CD8+ counts suggests that the cytokine response may dampen adaptive immunity against COVID‐19 infection,^10^ which is associated with atrophy of the secondary lymphoid tissues.^7^ Anti‐inflammatory treatment has been proposed but challenged with the dilemma of balancing the risk of secondary infection.^11^

Mesenchymal stromal cells (MSCs, also known as mesenchymal stem cells and medicinal signaling cells) are characterized by the presence of cell surface markers—CD44, CD90, CD105—and the absence of hematopoietic markers—CD34, CD45—as well as HLA‐DR. MSCs, which can be identified in diverse tissues, are most commonly sourced from bone marrow, adipose tissue, or umbilical cord. For translational research, MSCs are categorized into different generations according to their preparation strategy as minimally manipulated (G1), culture‐expanded (G2), lineage‐induced (G3), or genetically modified (G4).^12^

MSCs have been studied as a promising candidate to treat certain inflammatory conditions and immunologic diseases based upon their well‐characterized immunomodulatory effects, especially in the treatment of graft‐vs‐host disease, where MSC therapy was found to substantially improve complete response and overall survival.^13^, ^14^ The immunomodulatory activities are thought to include (a) inhibition of the proliferation and function of T cells, B cells, dendritic cells, and natural killer cells; (b) monocyte polarization to anti‐inflammatory M2 macrophages; and (c) production of IL‐10 and decreased production of TNF‐α and IL‐12.^15^, ^16^, ^17^ In addition, MSCs have powerful antifibrotic effects and may alleviate lung fibrosis.^18^, ^19^

An urgent question is whether large‐scale clinical trials and compassionate use of MSC therapy should be instituted to treat COVID‐19‐induced ARDS. To address this question, we systematically reviewed the available literature on safety, efficacy, and cytokine responses to MSC therapies in patients with ARDS.

## 2 METHODS

The study protocol was finalized at the beginning of the project, which defined objectives, search strategy, inclusion/exclusion criteria, data extraction, outcomes of interest, and analytical approaches.

### 2.1 Search strategy

A comprehensive search of databases from 1990 to March 31, 2020, in any language, was conducted. The databases included Ovid MEDLINE(R) and Epub Ahead of Print, In‐Process & Other Non‐Indexed Citations, and Daily, Ovid EMBASE, Ovid Cochrane Central Register of Controlled Trials, Ovid Cochrane Database of Systematic Reviews, Scopus, and ClinicalTrials.gov. The search strategy was designed and conducted by a medical reference librarian with input from the investigators. Controlled vocabulary supplemented with keywords was used to search for cell‐based therapy for COVID‐19 pneumonia. The actual strategy, listing all search terms, is available in the Appendices.

### 2.2 Eligibility criteria

We included randomized controlled trials (RCTs), observational studies, case reports, and case series that evaluated safety and/or efficacy of stem cells administered to adult patients with a diagnosis of COVID‐19 pneumonia or ARDS from any cause. MSCs that were culture‐expanded or minimally manipulated were included. We included studies regardless of language of publication. Studies were excluded if they did not report original data (eg, clinical reviews, editorials, letters, or erratum).

### 2.3 Outcome measures

The primary outcome was safety based on the frequency of serious adverse events (SAEs), adverse events (AEs), and if they were related to the treatment with MSCs.

Other outcomes of interest were clinical and laboratory findings, including mortality, level of oxygenation, lymphopenia, PaO_2_/FiO_2_ ratio, FEV1, FVC, FEV1/FVC, FEF50%, ventilator‐free days, ICU‐free days, organ failure‐free days, inflammatory markers including IL‐1, IL‐6, IL‐8, RAGE, TNF‐α, and CRP, angiotensin II, IL‐10. Radiographic and computed tomographic (CT) findings were also included.

### 2.4 Study selection process

**FIGURE 1:**
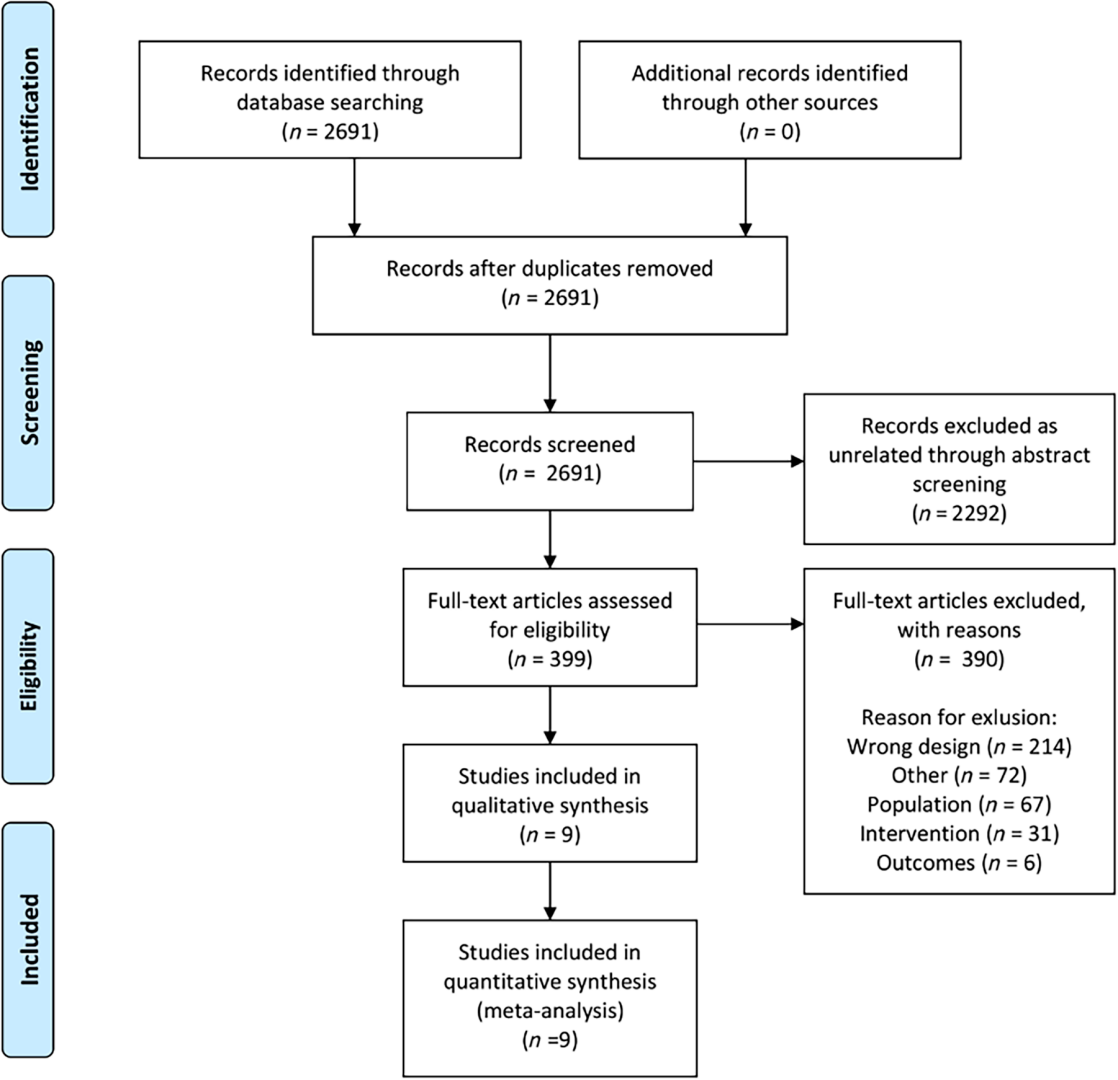
PRISMA 2009 flow diagram

From all citations received through the extensive literature search, duplicate studies were removed. Titles and abstracts were screened for inclusion by pairs of independent reviewers using DistillerSR (Evidence Partners, Ottawa, Canada) (E. K., E. E.‐C., V. P. S., J. C. H., M. E. W.). Relevant abstracts were recorded and full text articles were retrieved and screened by the same pairs of independent reviewers. Disagreements were resolved by a third independent reviewer (W. Q.) (Figure 1).

### 2.5 Data collection

A standardized data extraction form was developed and piloted. We extracted publication characteristics, study population, intervention and comparison details, and outcomes measures.

### 2.6 Risk of bias/certainty in evidence

For RCTs, we used the Cochrane risk of bias tool to evaluate bias from sequence generation, allocation concealment, blinding of participants, personnel, and outcome assessor, incomplete outcome data, selective outcome reporting, and other sources of bias. For observational studies, case series, and case reports, we used the Newcastle‐Ottawa Scale to evaluate representativeness of study population, ascertainment of exposure, comparability between groups (if applicable), outcome data source, and blinding of outcome assessment (Appendix 1, Tables 1 and 2). We used the GRADE approach (Grading of recommendations, development assessment and Evaluation) to rate certainty in the effect of MSC on mortality.^29^

**TABLE 1:** Patient characteristics

Author	Country	Study type	Total, n MSC; Ctrl	Mean age(y) MSC; Crtl	Comorbidities MSC; Ctrl	Baseline pulmonary function MSC; Ctrl	Baseline general MSC; Ctrl	F/U	ARDS cause	ARDS severity
Leng et al^20^	China	Phase I	10 (7; 3)	57; 65	HT (1); NR	Sat. 92% (0.02) SOB 2.29 (scale 1‐3, SD 0.95); Sat 92% (0.01) SOB 2 (scale 1‐3, SD 1)	Temp. 38.48°C (0.46); Temp. 37.53°C (1.46)	14D	COVID‐19	NR
Wilson et al^21^	United States	Phase I	9 (9; ‐)	NR; NA	NR; NA	Tidal vol. 6.2 (0.51) mL/kg PBW, PP 25.125 (9.79) cm H_2_O, PEEP 10.22 (1.56) cm H_2_O, PaO_2_/FiO_2_ 141.88 (33.90) mmHg, LIS 2.87 (0.40); NA	APACHE III 108.1 (24.4)	6M	Pneumonia, aspiration	Moderate
Zheng et al^22^	China	Phase I RCT	12 (6; 6)	66.7; 69.8	HT (3), CAD (1), neurologic disease (5), COPD (1), DM2 (2); HT (1), CAD (1), neurologic disease (3), COPD (0), DM (1)	PaO_2_/FiO_2_ 122.4 (42.0), RR 30.7 (3.3); PaO_2_/FiO_2_ 103.5 (32.2), RR 34 (6.2)	APACHE II 27.2 (6.4), pH 7.42 (0.08); APACHE II 23.0 (5.1), pH 7.36 (0.24)	28D	Pneumonia, aspiration	NR
Bellingan et al^23^	United Kingdom, United States	Phase I/II	36 (26; 10)	51; 59	NR; NR	PaO_2_/FiO_2_ 173 mmHg; PaO_2_/FiO_2_ 128 mmHg	SOFA scores 10.9 (2.2), vasopressor support 45%; SOFA 12.2 (4.2), vasopressor support 30%	28D	NR	Moderate‐severe
Matthay et al^24^	United States	Phase II RCT	60 (40; 20)	55; 55	NR; NR	MV (l/min) 11.1 (3.2), RR 27.8 (6.60), TV (cm H_2_O) 6.3 (0.9), MAP (cm H_2_O) 17.8 (4.9), PAP (cm H_2_O) 26.4 (5.7), PEEP cm H_2_O 12.4 (3.7), driving pressure (cm H_2_O) 14.0 (4.1), PaO_2_/FiO_2_ kPa 18.1 (4.3), oxygenation index (kPa) 98.7 (78.1‐123.3), LIS 3.1 (0.4); MV (l/min) 9.6 (2.4), RR 24.5 (6.3), TV (cm H_2_O) 6.1 (0.7), MAP (cm H_2_O) 16.4 (3.6), PAP (cm H_2_O) 23.7 (5.1), PEEP (cm H_2_O) 10.8 (2.6), driving pressure (cm H_2_O) 12.5 (4.3), PaO_2_/FiO_2_ (kPa) 19.1 (5.2), oxygenation index (kPa) 95.6 (71.6‐113.8), LIS 3.0 (0.5)	SOFA 8.1 (3.3), APACHE III 104 (31); SOFA 6.9 (2.7), APACHE III 89 (33)	12M	Sepsis, pneumonia, aspiration	Moderate‐severe
Yip et al^25^	Taiwan	Phase I dose escalation	9 (9; ‐)	54; ‐	HT (33.3%), DM‐2 (33.3%) dyslipidemia (22.2%), CHKD (55.6%), chronic liver dis. (22.2%), heart failure (11.1%), AKI; N/A	PaO_2_/FiO_2_ 108; NA	RR 24, Sepsis or multiorgan dysfunction 67%, dysfunctional organ # 3.1; fulminant myocarditis 22%; NA	NR	Pneumonia + other	Moderate‐severe
Chen et al^26^	China	Phase I/II nonrandomized	61 (17; 44)	62.8; 61.6	HT (58.8%), CAD (0%), COPD (0%), DM2 (29.4%), liver disease (5.9%), renal failure (9%); HT (52.3%), CAD (18.2%), COPD (2.3%), DM2 (15.9%), liver disease (2.3%), renal failure (22.7%)	Mech. ventilation 14 (82.4%), ECMO 8 (47.1%); Mech. ventilation 31 (70.5%), ECMO 14 (31.81%)	ALSS 13 (76.5%), CRRT 12 (70.6%); ALSS 18 (40.9%), CRRT 16 (36.4%)	5Y (4 pts), 12 m o.	H7N9	NR
Chang et al^27^	South Korea	Case report	1 (1; ‐)	59; ‐	ITP (on corticosteroids); pulmonary fibrosis; N/A	Initial SaO_2_ level was 75% and his PaO_2_/FiO_2_ ratio was 166 mmHg; NA	NR	118D	Pneumonia	NR
Simonson et al^28^	Sweden	Case series	2 (2; ‐)	49; ‐	HT (50%), AML (50%); N/A	P1: diffuse bilat. infiltrates, progressive resp. failure requiring mech. ventilation, followed by VV, and later by VA ECMO, AKI, requiring CRRT. P2: mechanical ventilation, bilat. infiltrates. neutropenic, VV ECMO, severe transfusion‐dependent cytotoxic chemotherapy‐induced thrombocytopenia, CRRT	NR	2M	Infection	Severe

Abbreviations: AKI, acute kidney injury; ALSS, artificial support liver system; AML, acute myeloid leukemia; APACHE, Acute Physiology and Chronic Health Evaluation; ARDS, acute respiratory distress syndrome; BM‐MSCs, bone marrow derived mesenchymal stem cells; CAD, coronary artery disease; CHKD, chronic kidney disease; COPD, chronic obstructive pulmonary disease; CRRT, continuous renal replacement therapy; D, day; DM, diabetes mellitus; ECMO, extracorporeal membrane oxygenation; F/U, follow‐up; HT, hypertension; ITP, idiopathic thrombocytopenic purpura; LIS, lung injury score; M, month; MAP, mean airway pressure; MSCs, mesenchymal stem cells; MV, minute ventilation; NA, not applicable; NR, not reported; P, patient; PaO2/FiO2, arterial oxygen partial pressure/fractional inspired oxygen; PAP, plateau airway pressure; PBW, predicted body weight; PEEP, positive end‐expiratory pressure; Pt, patients; PP, plateau pressure; RCT, randomized controlled trial; RR, respiratory rate; SOB, shortness of breath; SOFA, sequential organ failure assessment; TV, tidal volume; VA, veno‐arterial; VV, veno‐venous; Y, years.

**TABLE 2:** Product characterization

Author	MSCs source^a^	Donor n (gender)	Surface markers	Passage	Culture media	MSC dose per kg	Viability	Frequency	Route of delivery	Control
Chang et al^27^	Umbilical cord blood	1 (NR)	Positive CD29, CD44, CD73, CD105, CD166, and negative CD34, CD45, CD14, and HLA‐DR	6	NR	1 × 10^6^	NR	1	Intratracheal	No control group
Simonson et al^28^	Bone marrow	1 (M)	Positive CD73, CD90, CD105, HLA‐ABC, and negative CD14, CD31, CD34,CD45, and HLA‐DR	NR	DMEM‐low glucose supplemented with lysed human platelets	2 × 10^6^	95%	1	Central venous catheter	No control group
Yip et al^25^	Umbilical cord, Wharton's jelly	NR	NR	NR	NR	1.0 × 10^6^ 5.0 × 10^6^ 1.0 × 10^7^	NR	1	IV	No control group
Leng et al^20^	NR	NR	NR	NR	NR	1 × 10^6^	NR	1	IV	NR
Wilson et al^21^	Bone marrow	1 (M)	NR	NR	NR	1 × 10^6^, 5 × 10^6^, or 10 × 10^6^	50%‐63% (mean 56%)	1	IV	No control group
Zheng et al^22^	Adipose tissue	1 (F)	CD73, CD90, CD105, CD34, CD45, HLA‐DR analyzed	Max.4	DMEM‐low glucose + penicillin, streptomycin, 2% FBS, EGF, and FGF	1 × 10^6^	NR	1	IV	100 mL normal saline
Chen et al^26^	Menstrual blood	1	NR	NR	NR	Total dose: 100 mL	NR	3 (9 pts) 4 (8 pts)	IV	NR
Bellingan et al^23^	Bone marrow	NR	NR	NR	NR	Total dose: 300 × 10^6^ (3 pts); Total dose: 900 × 10^6^ (23 pts)	NR	1	IV	NR
Matthay et al^24^	Bone Marrow	3 (1 F, 2 M)	CD105, CD73, CD90, CD45, CD34, CD14, CD19, and HLA‐DR tested	NR	NR	10 × 10^6^	≥70%	1	IV	Plasmalyte A, 100 mL

Abbreviations: DMEM, Dulbecco's modified Eagle's medium; EGF, epidermal growth factor; F, female; FBS, fetal bovine serum; FGF, fibroblast growth factor; IV, intravenous; M, male; MSCs, mesenchymal stem cells; NA, not applicable; NR, not reported; pts, patients.

a All MSCs were allogeneic and culture‐expanded.

### 2.7 Statistical analysis

We conducted meta‐analysis to quantitatively summarize study findings based on the similarities of PICOTS (patient, intervention, control, outcome, timing, setting) presented by the studies. Mortality was the only outcome deemed to be appropriate for meta‐analysis. The DerSimonian‐Laird random effect model with Hartung‐Knapp‐Sidik‐Jonkman variance correction^30^ was used to pool odds ratio (OR) from the included studies. We used I2 indicator to evaluate heterogeneity between studies. Subgroup analyses were conducted based on study design (RCTs vs observational studies). We were unable to evaluate publication bias due to the small number of available studies. All statistical analyses were conducted using Stata version 16.1 (StataCorp, LLC, College Station, Texas).

## 3 RESULTS

The literature search identified 2691 unique citations. Abstract and full‐text screening identified nine studies with 200 patients to be included for the data extraction. Full text exclusion reasons are presented in Table 3.

**TABLE 3:** Analysis inclusion and exclusion criteria

PICOTS elements	Inclusion criteria	Exclusion criteria
Population	Patients with any coronavirus pneumonia (COVID‐19, SARS1, MERS, others) Patients with acute respiratory distress syndrome (ARDS), acute respiratory distress syndrome, acute respiratory failure Adults 18 y and older	Animals Children (age <18 y)
Interventions	KQ: mesenchymal stem cell therapy transplantation, include: Stem cell Mesenchymal stem cell Mesenchymal stromal cell MSCs/MSC Progenitor cell iPS iPSC	
Comparators	Usual care, supportive care only, no treatment	None
Outcomes	Mortality, CT, time to hospital discharge, time to recovery, lymphocyte count, admission to ICU, needs for intubation, adverse events	None
Timing	Any time	None
Settings	Any setting (inpatient, outpatient, emergency department)	None
Study design	Any study design (including case reports) Any sample size	In vitro studies, nonoriginal data (eg, narrative reviews, editorials, letters, or erratum), qualitative studies, cost‐benefit analysis, cross‐sectional (ie, nonlongitudinal)
Publications	Any language 1990 to March 31, 2020	

Abbreviations: ARDS, acute respiratory distress syndrome; CT, computed tomography; ICU, intensive care unit; MSC, mesenchymal stromal cell.

### 3.1 Study characteristics

Of the nine studies, there were four phase I clinical trials, three phase I/II or phase II clinical trials, one case report, and one case series. Five were comparative studies with control groups, including three RCTs. All nine studies reported SAEs, AEs, mortality, and pulmonary function outcomes (n = 200). Six studies evaluated inflammatory markers (n = 98). A total of 117 patients received MSC therapy, while 83 participated as controls (Table 1). The risk of bias of included randomized trials was high due to unclear random sequence generation and allocation concealment procedures.

### 3.2 Patient characteristics

The studies were from six countries and regions, including China (n = 3),^20^, ^22^, ^26^ the United States (n = 2),^21^, ^24^ United States/United Kingdom (n = 1),^23^ Sweden (n = 1),^28^ Taiwan (n = 1),^25^ and South Korea (n = 1).^27^ The average age of study participants was 51 to 67 years for the MSC groups and 55 to 70 years for the control groups in the comparative studies. Baseline PaO_2_/FiO_2_ was in a range of 100 to 200 in six studies^21^, ^22^, ^23^, ^24^, ^25^, ^27^ and not reported in two studies in which all patients were on ventilator or extracorporeal membrane oxygenation (ECMO) support.^26^, ^28^ Baseline Acute Physiology and Chronic Health Evaluation (APACHE) scores were between 23 and 27 in one study,^22^ averaged 108 in one study,^21^ and 104 in the MSC group vs 89 in the control group in one study^24^ (Table 1).

### 3.3 Intervention characteristics

Culture‐expanded allogeneic MSCs were used in all nine included studies. Allogeneic bone marrow‐derived MSCs were used in four studies,^21^, ^23^, ^24^, ^26^ adipose‐derived MSCs in one study,^22^ menstrual blood MSCs in one study,^26^ umbilical cord Wharton's jelly‐derived MSCs in one study,^25^ and umbilical cord blood MSCs in one study.^27^ One study did not report the tissue origin of the MSCs and described their cell‐product as commercially obtained^20^ (Table 2).

The MSCs used in the included studies were each from a different manufacturing facility. Characterization was reported in some studies, with a significant variability of detail. MSCs from one donor were used in five studies,^22^, ^24^, ^26^, ^27^, ^28^ from three donors in one study,^24^ and not specified in three studies.^20^, ^23^, ^25^ Characterization of MSCs was reported in four studies, with most of the following markers: positive for CD29, CD44, CD73, CD90, CD105, CD166, HLA‐ABC, and negative for CD31, CD34, CD45, CD14, and HLA‐DR.^22^, ^24^, ^25^, ^28^ Viability was reported to be 50% to 95% (Table 2).^21^, ^24^, ^28^ In addition, MSCs were found to be ACE2‐ or TMPRSS2‐negative, indicating that these MSCs were unlikely to become infected by COVID‐19.^20^

MSCs were administered intratracheally (n = 1),^27^ intravenously (n = 115), or through a central venous catheter (n = 1).^20^, ^21^, ^22^, ^23^, ^24^, ^25^, ^26^, ^28^ MSC infusion frequency ranged from a single administration in eight studies^20^, ^21^, ^22^, ^23^, ^24^, ^25^, ^27^, ^28^ to 3 to 4 administrations in one study.^26^ Dosing of the stem cells ranged from 1 to 10 million cells per kilogram of body weight or a uniform dose of 300 or 900 million cells.^23^ In one study, dosing was not specified^26^ (Table 2). Patients in control groups were treated with placebo, including saline^22^ or Plasmalyte A,^24^ or standard of care.

### 3.4 Primary outcome: safety

#### 3.4.1 Serious adverse events

There were no reported treatment‐related SAEs. One patient experienced an unexpected SAE described as multiple organ embolic infarcts, which was thought to be present before the MSC treatment as noted on a previously performed magnetic resonance imaging scan. Across all studies, none of the 66 deaths were categorized as related to the MSC therapy (Table 4).

**TABLE 4:** Adverse events and serious adverse events

Author	AEs treatment‐related	SAEs nontreatment‐related	SAEs treatment‐related
Leng et al^20^	No acute infusion‐related or allergic reactions were observed within 2 h after transplantation, no delayed hypersensitivity or secondary infections detected	None	None
Wilson et al^21^	None	Two patients expired 7 d after the MSCs infusion, and one patient was discovered to have multiple embolic infarcts of the spleen, kidneys, and brain that were age‐indeterminate and thought to have occurred before the MSCs infusion based on MRI results	None
Zheng et al^22^	One patient from each group had diarrhea, one had rash after infusion that resolved within 24 h, resolved within 48 h. One patient in the MSCs group developed rash in the chest area	3 deaths: 1 in the MSCs group; 2 in the placebo group	None
Bellingan et al^23^	1 CTCAE grade‐1 infusion‐related reaction, reported as possibly related to cell treatment, resolved without intervention	5 deaths in the MSCs group, 4 in the placebo group	None
Matthay et al^24^	None	One patient in the MSCs group died within 24 h of MSCs infusion; death was judged to be probably unrelated; otherwise 60‐d mortality was 15 patients in the MSCs group and 5 in the placebo group	None
Yip et al^25^	Transient desaturation, dyspnea, and hypotension at 10‐15 min after cell infusion were observed in two cases, one patient had generalized skin rash persistent for 2 d	3 deaths	None
Chen et al^26^	None	1 death in MSC group	None
Chang et al^27^	NR	1 death	None
Simonson et al^28^	None	None	None

Abbreviations: AE, adverse events; CTCAE, Common Terminology Criteria for Adverse Events; MRI, magnetic resonance imaging; MSCs, mesenchymal stromal cells; NR, not reported; SAE, serious adverse events.

#### 3.4.2 Adverse events

Mild AEs were reported. One patient experienced a grade I allergic reaction.^23^ In another study, patients experienced diarrhea in both treatment groups.^22^ Yip et al^25^ reported transient desaturation, dyspnea, and hypotension shortly after cell infusion in two patients, and one patient had a generalized skin rash that persisted for 2 days. All reported AEs resolved spontaneously (Table 4).

### 3.5 Secondary outcome: efficacy

#### 3.5.1 Mortality

**FIGURE 2:**
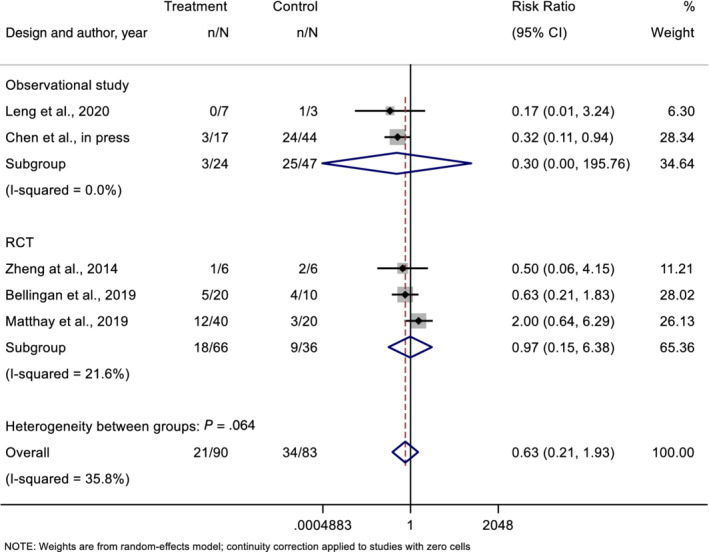
Pooled estimate for mortality

Mortality was reported in all studies. Comparisons between treatment and control groups were made in five studies (n = 179).^20^, ^22^, ^23^, ^24^, ^26^ In four of five studies, there was a trend toward a decreased mortality rate in the MSC treatment group. In the one study that showed an increased mortality rate, the authors noted that at baseline the MSC treatment group had greater severity of illness as evidenced by higher baseline APACHE and sequential organ failure assessment (SOFA) scores.^24^ The overall mortality rates were 25% (24 of 96) for MSC‐treated patients and 43% (36 of 83) in controls. Although there was a favorable trend, the overall difference did not reach statistical significance (Peto OR 0.63, 95% confidence interval 0.21‐1.93). The certainty in evidence about an effect on mortality was low, due to imprecision and methodological limitations of the included trials, as presented in Figure 2 and Table 5.

**TABLE 5:** Clinical, laboratory, and imaging outcomes

Author	Mortality MSCs (death/n)	Mortality in control (death/n)	Pulmonary function outcomes	Systemic outcomes	Inflammatory markers	Imaging
Leng et al^20^	0/7	1/3	At 2‐4 d after MSC transplantation, all symptoms disappeared in all patients, oxygen saturations rose to ≥95% at rest, with or without oxygen uptake (5 L/min)	At 2‐4 d after MSC transplantation, all symptoms disappeared in all patients	MSC group: Decrease in TNFα, increase in IL‐10 (all group), most sick pt: CRP decreased; cytokine‐secreting immune cells CXCR3+CD4+ T cells, CXCR3+CD8+ T cells, and CXCR3+ NK cells;, IP‐10, VEGF, lymphocytes, D14+CD11c+CD11bmid regulatory DC cell population dramatically increased	The patient in the most critical condition had signs of pneumonia and ground glass opacity on CT improvement on D9 after MSCs given.
Wilson et al^21^	2/9	NA	Mean LIS improved between baseline and D3 in all three dosing groups; there was dose‐dependent trend; no pt received ECMO, 2/9 were extubated before D3 (not statistically significant)	Mean SOFA score: declined in all three dosing groups over the first 3 d	Median levels of IL‐6, IL‐8, RAGE declined between baseline and D3. Between group comparison *P* = .3679, .3189, .3189, and .8669, respectively	NR
Zheng et al^22^	1/6	2/6	Ventilator‐free days and ICU‐free days at D28 after treatment were similar in both groups PaO_2_/FiO_2_ did not differ significantly between MSCs and placebo groups at all time points	Length of hospital stay, at D28 after treatment were similar	MSC group: serum SP‐D levels at day 5 were significantly lower than those at day 0. Changes in IL‐8 levels not significant. IL‐6 levels at day 5 showed a trend toward lower levels as compared with day 0, but not statistically significant	NR
Bellingan et al^23^	5/26	4/10	ICU‐free days 10.3 ± 8.9, ventilator‐free days 12.9 ± 10.7 in MSCs group and 8.1 (8.9) and 9.2 (9.6) in the control group, respectively	NR	NR	NR
Matthay et al^24^	15/40 (to day 60)	5/20 (to day 60)	Mortality higher in the MSC group (thought to be due to higher APACHE baseline score in the MSCs group), ventilator‐free and organ‐failure free days were all lower in the MSC group than in the placebo group (not significant). At 2 d post‐transplantation, oxygen index in the MSC group reduced (not significant) more than in the placebo group (oxygenation similar at baseline)	Number of ventilator‐free and organ failure‐free days were all lower in the MSC group than in the placebo group, but the differences were not significant; number of intensive‐care‐free days was higher in the placebo group than in the MSC group	Concentrations of angiopoietin 2 had reduced by 6 h after the start of infusion in the MSC group. No changes from baseline were seen for IL‐6, IL‐8, RAGE, or protein C concentrations at 6 or 24 h in either group	NR
Yip et al^25^	3/9	NA	Trend of improvement in PaO_2_/FiO_2_, ventilator‐free days was 12.9 (10.7) and in ICU‐free days was 10.3 (8.9)	Two patients who died expressed initial dramatic clinical improvement after MSC infusion for 3 d; another pt was improving and weaning off the ventilator but expired due to severe septic shock	Serial flow‐cytometric analyses of circulating inflammatory biomarkers were progressively reduced. The immune cell markers were notably increased after cell infusion.	Lobar consolidation improvements noted, 2 pts complete resolution, 3 pts progressive improvements, 3 mixed results
Chen et al^26^	3/17	24/44	Four MSC patients followed up for 5 y. No long‐term significant difference among pts in the resp. functions (FEV1, FVC, FEV1/FVC, and FEF 50%)	After following up for 2 y, the scores for all elements of the SF‐36 did not significantly differ during the follow‐up	NR	After MSC transplantation for 24 wk and 1 y, all patients showed improvement on CCT
Chang et al^27^	1/1	NA	D1 improvement from 191 to 334 (334 on D3) in PaO_2_/FiO_2_, improved inspiratory pressure from 26 to 24 mmHg, dynamic compliance improved from a preprocedure value of 22.7 to 26.5, 27.3, and 27.9 mL/cmH_2_O after 24, 48, and 72 h, respectively	Mental status, lung compliance, P/F ratio, chest radiography all showed improvement over the course of at least 3 d	NR	Chest radiography slight decrease in bilateral infiltrates
Simonson et al^28^	0/2	NA	Patient 1: pulmonary compliance increased from 6 to 20 mL/cm H_2_O at 2 d, to 44 mL/cm H_2_O at day 8, tidal volume from 100‐420 mL at D2, 720 mL at D5 Patient 2: pulmonary compliance increased from 20 mL/cm H_2_O to 35 mL/cm H_2_O at D1, to 73 mL/cm H_2_O at D12. Tidal volumes increased	In both patients: improved with resolution of respiratory, hemodynamic, and multiorgan failure	In both patients: BAL surfactant protein B increased (better alveolar‐epithelial integrity, pro‐inflammatory microRNAs significantly declined within 24 h of infusion, Pt1: decrease in epithelial apoptosis (ccK18, K18): IL‐6 and albumin levels in BAL fluid decreased; IL‐8 and IFN‐g in plasma initially decreased after injection	Serial CXRs demonstrated progressive decreases in pulmonary infiltrates at 24 h after infusion in both patients

Abbreviations: APACHE, Acute Physiology and Chronic Health Evaluation; BAL, bronchoalveolar lavage; CCT, cardiac computerized tomography; CT, computed tomography; CXR, chest X‐ray; D, day; ECMO, extracorporeal membrane oxygenation; ICU, intensive care unit; LIS, lung injury score; MSC, mesenchymal stromal cell; NA, not applicable; NR, not reported; PaO2/FiO2, arterial oxygen partial pressure/fractional inspired oxygen;pt, patient; SF‐36, 36‐Item Short Form Survey; SOFA, sequential organ failure assessment score.

#### 3.5.2 Pulmonary function changes

All studies reported pulmonary function tests as outcome measures. Lung injury score, tidal volumes, lung compliance, PaO_2_/FiO_2_, oxygenation, dependence on mechanical ventilators or ECMO, or ICU stay were found to improve within 5 days in most of the studies, while other studies found no long‐term differences in a 5‐year follow‐up. Imaging studies reported in five studies^20^, ^25^, ^26^, ^27^, ^28^ all showed post‐treatment improvement on CT or chest x‐ray (Table 5).

#### 3.5.3 Systemic changes and symptoms

Systemic changes were reported as any changes to overall patient status, including mental status, SOFA score, quality of life (SF‐36), laboratory markers of organ function, symptoms, and relative improvements from baseline (Table 5).^20^, ^21^, ^22^, ^24^, ^25^, ^26^, ^27^, ^28^

#### 3.5.4 Inflammatory cytokines

Inflammatory markers and cytokines were evaluated in six studies. These showed clear decreases in pro‐inflammatory cytokines, including IL‐1, IL‐6, TNF‐α, and CRP, and increased lymphocytes and IL‐10 within 5 days of MSC therapy (Table 5),^20^, ^21^, ^22^, ^24^, ^25^, ^28^ which supported an immunomodulatory effect of the MSC therapy.

## 4 DISCUSSION

### 4.1 Main findings

This systematic review examined the published results of MSC therapy for patients with ARDS, with focused analysis on safety, efficacy, and related immunologic and pulmonary responses. ARDS has been identified as a major mortality risk factor in COVID‐19 infected patients, with current projections of 50 000 to 100 000 deaths in the United States alone.^31^ Several repurposed and novel therapies for COVID‐19 are being examined that include targeting the virus, enhancing host immunity, and reducing organ damage. Numerous clinical studies have demonstrated that MSC therapy is safe and has the potential to mitigate inflammatory and physiologic damage for a variety of conditions involving the central nervous,^32^, ^33^ cardiac,^34^, ^35^ renal,^36^ gastrointestinal,^37^, ^38^, ^39^ and respiratory^40^, ^41^ systems. Our analysis of the literature suggests similar results for MSC therapy for treating ARDS in COVID‐19 patients.

Safety is the most important concern for all new therapies, especially in patients at high risk for death from the condition being treated, and was carefully evaluated for MSC‐treated patients in the studies reviewed. Of the 200 patients with ARDS that were treated with intravenously or intratracheally administered MSCs or placebo, 66 patients died, of which 30 were in the active treatment group. None of these 30 deaths were found to be related to MSC therapy, nor were any other SAEs attributed to the MSC therapy. Transient AEs were reported, but all of them resolved spontaneously in the short term. This safety profile is consistent with the experience of other human clinical trials involving MSC therapy.^33^, ^37^, ^38^, ^39^, ^40^, ^41^, ^42^, ^43^

The potential efficacy of MSC therapy for ARDS in COVID‐19‐infected patients was examined in this project considering death as the primary outcome. Of five controlled studies of MSC therapy in patients with ARDS, four reported a numeric reduction in mortality. In the one study showing higher mortality in MSC group, the MSC group had more severe baseline illness, which may have masked the efficacy of MSC treatment. If that study is excluded from the analysis, the mortality was 49% (31/63) in control patients compared with the 16% (9/56) in MSC‐treated patients. While not statistically significant, the difference suggests MSC therapy may be effective in reducing mortality in these patients.

Other outcomes examined included pulmonary function, systemic outcomes, and immune responses. Pulmonary function was evaluated in seven studies. Despite the heterogeneous nature of measures used in different studies, six reports showed improvement in pulmonary function within 3 to 5 days, and one study reported no change. Of the reported improved cases, there were notable improvements in lung compliance and tidal volumes. Imaging findings were reported in five studies, showing improvement of opacity in chest radiograph and chest CT within days after treatment. ICU‐ and ventilator‐free days were reported in three studies and had mixed results of improvement or no change in MSC treated patients compared to controls. These results showing improved pulmonary function and imaging findings further support consideration of MSC treatment for COVID‐19‐induced ARDS.

Findings from this study also suggest the impact of MSC therapy on important immunologic and inflammatory processes that lead to organ injury in COVID‐19‐infected patients. Cytokine release syndrome is a major underlying pathophysiological process in ARDS and was found to decrease with MSC therapy in seven of the studies analyzed.^20^, ^21^, ^22^, ^23^, ^24^, ^25^, ^28^

In addition, MSCs have been hypothesized to neutralize free virus particles through the production of antibiotic proteins like LL37, which bind to virus and lung cell binding sites.^42^

As recently reported, COVID‐19 not only affects the lung, but also the heart and kidney with reported cardiomyopathy and kidney injury.^43^, ^44^ Two of the analyzed studies reported improved resolution of multiple organ failure or increased organ failure‐free days with MSC treatment, which further supports their consideration for clinical use. Future clinical trials should include multiorgan failure parameters.

Senior age is associated with higher rate of mortality and has been reported to be associated with higher risk of death in patients with COVID‐19.^45^, ^46^ In comparative studies included in this review, the age ranges were similar in both the MSC and the control groups at baseline and appeared to have minimal impact on outcomes.

### 4.2 Clinical and research implications

This comprehensive systematic review of published reports of MSC therapy for ARDS has yielded several important findings. First, a significant number of individuals with ARDS (117) have been treated with MSC without evidence of SAEs. This finding, along with the similar safety profile for many other patients treated with MSC for a variety of conditions in the United States and globally, provides evidence of low risk. Second, MSC treatment was found to have a suggested benefit in reducing death and improving pulmonary function in patients with ARDS. Third, MSC treatment had a suggested benefit in mitigating the physiologic and immunologic responses leading to ARDS, supporting the potential mechanism of action, which has also been demonstrated with MSC therapy for other inflammatory disorders.

Since the closing date of this project (March 30, 2020), we have learned that patients in Israel have received MSC treatment under compassionate use with apparent beneficial results.^47^, ^48^ A study performed in the United States by industry has shown improved survival rate in preliminary results.^49^ In addition, from personal communications, MSCs had been used in six patients at another site in the United States by April 26, 2020.

Therefore, we believe that the current evidence suggests a potential favorable risk‐benefit ratio and mechanistic justification to continue compassionate use and initiate large‐scale trials of MSC therapy for COVID‐19 patients in the United States, using MSCs that have already demonstrated safety in FDA‐approved studies. Multicenter clinical trials should be conducted with MSCs characterized according to standard protocol.

### 4.3 Limitations

The limitations of this systematic review include the lack of large‐scale RCTs, the heterogeneity of outcome measures on pulmonary function, and the lack of studies collecting extra‐pulmonary pathology. In addition, variability of MSC products prevents the assessment of which MSC type or preparation may be effective. Moreover, there may exist selection bias in published results and case reports that favors positive findings for MSC therapy for ARDS vs results that were equivocal or negative and not published.

## 5 CONCLUSION

This analysis of published reports demonstrated the potential benefits, minimal risks, and presumptive mechanisms of MSC therapy for ARDS, which support the rationale for treatment of COVID‐19 patients with pulmonary disease. Adequately powered clinical trials are urgently needed to test clinical outcomes in patients with COVID‐19 syndrome and SARS‐CoV‐2 infection, and should use well‐characterized MSC products with documented safety profiles from FDA‐approved studies.

## CONFLICT OF INTEREST

W. Q., J. M. H., J. K., and J. M. M. reported roles as principal investigators of MSC trials. F. P. S. is a paid consultant as Medical Director of The Marcus Foundation, which funds numerous clinical trials involving MSCs. J. M. M. is an unpaid Scientific Advisor for BreStem Therapeutics. J. M. H. reported having a patent for cardiac cell‐based therapy, holds equity in Vestion, Inc., and maintains a professional relationship with Vestion, Inc. as a consultant and member of the Board of Directors and Scientific Advisory Board; J. M. H. is also the Chief Scientific Officer, a compensated consultant and advisory board member, for Longeveron and holds equity in Longeveron; J. M. H. is the coinventor of intellectual property licensed to Longeveron. J. M. H. declared inventor or patent holder and research funding from Longeveron, Heart Genomics; advisory role and research funding with Vestion; research funding from NHLBI. J. K. declare Intellectual property rights with IDF, hCT‐MSC for treatment of ASD, HIE, CP; NMDP Scientific Advisor; Celularity SAB; research funding from the Marcus Foundation, NIH, HRSA; leadership position with Istari—CMO (spouse). F. P. S. declared advisory role with the Marcus Foundation, Neuvana, Radix Health. The other authors indicated no potential conflicts of interest.

## AUTHOR CONTRIBUTIONS

W.Q., E.K., E.E‐C.: conception and design, collection and/or assembly of data, data analysis and interpretation, manuscript writing, final approval of manuscript; Z.W.: conception and design, collection and/or assembly of data, data analysis and interpretation, final approval of manuscript; J.M.H., F.P.S.: conception and design, critical review of the manuscript, manuscript writing, final approval of manuscript; G.B., J.M.P., J.M.M., A.I.C., J.K., A.C.Z., M.H.M.: conception and design, critical review of the manuscript, final approval of manuscript; T.N., V.P.S., J.C.H., M.E.W., L.J.P.: collection and/or assembly of data, final approval of manuscript.

## Data Availability

Data sharing is not applicable to this article as no new data were created or analyzed in this study.

## APPENDIX A: RISK OF BIAS ASSESSMENT 1

**TABLE A1:** Risk of bias of randomized controlled trials^22^, ^23^, ^24^

Author	Year	Sequence generation	Allocation concealment	Participants and personnel blinding	Outcome assessment blinding	Incomplete outcome data	Selective reporting	Other sources of bias	Overall risk of bias
Bellingan et al	2019	Unclear	Unclear	Low risk	Unclear	Unclear	Unclear	Unclear	Moderate risk
Matthay et al	2019	Low risk	Low risk	Low risk	Low risk	Low risk	Low risk	Low risk	Low risk
Zheng et al	2014	Unclear	Unclear	Low risk	Unclear	Low risk	Unclear	Low risk	Moderate risk

**TABLE A2:** Risk of bias of observational studies, case series, and case studies^20^, ^21^, ^25^, ^26^, ^27^, ^28^

Author	Year	Representativeness of study cohort	Ascertainment of exposure	Comparability between groups	Outcome data source	Independent blind assessment of outcome	Overall risk of bias
Chang et al	2014	Low risk	Low risk	Not applicable	Low risk	High risk	Moderate risk
Simonson et al	2015	Low risk	Low risk	Not applicable	Low risk	High risk	Moderate risk
Yip et al.	2020	Low risk	Low risk	Not applicable	Moderate risk	High risk	High risk
Leng et al	2020	Low risk	Low risk	Moderate risk	Moderate risk	High risk	High risk
Wilson et al	2015	Low risk	Low risk	Not applicable	Low risk	Moderate risk	Moderate risk
Chen et al	2020	Low risk	Low risk	Moderate risk	Low risk	Low risk	Moderate risk

## APPENDIX B: ACTUAL SEARCH STRATEGY 1

### Ovid

Database(s): EBM Reviews—Cochrane Central Register of Controlled Trials February 2020, EBM Reviews—Cochrane Database of Systematic Reviews 2005 to March 25, 2020, Embase 1974 to March 30, 2020, Ovid MEDLINE(R) and Epub Ahead of Print, In‐Process & Other Non‐Indexed Citations and Daily 1946 to March 30, 2020.

Search strategy is given below.

### Scopus

1. TITLE‐ABS‐KEY(((“Corona virinae” or “corona virus” or Coronavirinae or coronavirus or COVID or nCoV or HCoV) W/4 (“19” or “2019” or novel or new or pneumonia*)) OR ((“Corona virinae” or “corona virus” or Coronavirinae or coronavirus or COVID or nCoV or HCoV) and Wuhan) OR “Acute Respiratory Distress Syndrome” OR ARDS OR “Corona virinae19” OR “Corona virinae2019” OR “corona virus19” OR “corona virus2019” OR Coronavirinae19 OR Coronavirinae2019 OR coronavirus19 OR coronavirus2019 OR COVID19 OR COVID2019 OR “cytokine storm*” OR “interstitial pneumonia*” OR MERS OR “Middle East Respiratory Syndrome” OR nCOV19 OR nCOV2019 OR “SARS Corona virus” OR “SARS Coronavirus” OR “SARS‐COV” OR “Severe Acute Respiratory Syndrome Corona virus” OR “Severe Acute Respiratory Syndrome Coronavirus” OR “viral pneumonia*” OR “virus pneumonia*”)
2. TITLE‐ABS‐KEY(“adipose derived stromal cell*” OR “adipose derived stromal stem cell*” OR “adipose stromal cell*” OR “adipose stromal stem cell*” OR “bone marrow derived stromal cell*” OR “bone marrow derived stromal stem cell*” OR “bone marrow stromal cell*” OR “bone marrow stromal stem cell*” OR “colony forming unit*” OR “mesenchymal cell*” OR “mesenchymal progenitor cell*” OR “mesenchymal stem cell*” OR “mesenchymal stromal cell*” OR “mother cell*” OR MSC OR MSCs OR “progenitor cell*” OR “stem cell*” OR “umbilical cord derived stromal cell*” OR “umbilical cord derived stromal stem cell*” OR “umbilical cord stromal cell*” OR “umbilical cord stromal stem cell*” OR “Wharton jelly cell*” OR “Wharton's jelly cell*”)
3. PUBYEAR AFT 1989
4. 1 and 2 and 3
5. INDEX(embase) OR INDEX(medline) OR PMID(0* OR 1* OR 2* OR 3* OR 4* OR 5* OR 6* OR 7* OR 8* OR 9*)
6. 4 and not 5

### ClinicalTrials.gov

Condition or disease

(“2019 novel coronavirus” OR “2019‐nCoV” OR “acute respiratory distress” OR “acute respiratory syndrome” OR ARDS OR Coronavirus OR “COVID 19” OR “cytokine storm” OR “interstitial pneumonia” OR MERS OR “Middle East Respiratory Syndrome” OR “SARS‐CoV”)

(“viral pneumonia” OR “virus pneumonia”)

Other terms

(“colony forming unit” OR “mesenchymal cell” OR “mother cell” OR MSC OR MSCs OR “progenitor cell” OR “stem cell” OR “stromal cell” OR “wharton jelly cell” OR “whartons jelly cell”)

First posting

January 1, 1990 to March 31, 2020
